# Hypercoagulability in Pulmonary Tuberculosis: Reduced Protein C and Free Protein S Predict Pulmonary Embolism—Evidence from a Prospective Romanian Cohort

**DOI:** 10.3390/jcm15051903

**Published:** 2026-03-02

**Authors:** Denisa Maria Mitroi, Silviu Gabriel Vlasceanu, Ovidiu Mircea Zlatian, Mihai Olteanu, Oana Maria Catană, Radu Razvan Mititelu, Anca Lelia Riza, Georgiana Camen, Viorel Biciușcă, Ramona Cioboată

**Affiliations:** 1 Doctoral School, University of Medicine and Pharmacy, 200349 Craiova, Romania; denisa_maria2@yahoo.com (D.M.M.); oana_cattana@yahoo.com (O.M.C.); 2 Department of Physiology, “Carol Davila” University of Medicine and Pharmacy, 050474 Bucharest, Romania; silviu.vlasceanu@drd.umfcd.ro; 3 Department of Thoracic Surgery, Marius Nasta Pneumology Institute, 050159 Bucharest, Romania; 4 Microbiology Department, University of Medicine and Pharmacy of Craiova, 200349 Craiova, Romania; radu.mititelu@umfcv.ro; 5 Pneumology Department, University of Medicine and Pharmacy, 200349 Craiova, Romania; biciuscaviorel@gmail.com (V.B.); ramona_cioboata@yahoo.com (R.C.); 6 Laboratory of Human Genomics, University of Medicine and Pharmacy of Craiova, 200638 Craiova, Romania; anca.costache@umfcv.ro; 7 Radiology Department, University of Medicine and Pharmacy of Craiova, 200638 Craiova, Romania; georgiana.camen@umfcv.ro

**Keywords:** pulmonary tuberculosis, protein C, free protein S, CT abnormalities, hypercoagulability, pulmonary embolism

## Abstract

**Background/Objectives:** Pulmonary tuberculosis (TB) is accompanied by inflammation-driven hypercoagulability and increased venous thromboembolism risk. We investigated whether the natural anticoagulants protein C and free protein S are reduced in active TB and whether baseline levels are associated with bacillary burden, treatment response, CT evolution, and pulmonary embolism (PE). **Methods**: We conducted a prospective cohort study in Romania, including 63 adults with newly diagnosed, bacteriologically confirmed, drug-susceptible pulmonary TB and 30 TB-free controls (October 2024–December 2025). Venous blood was collected at baseline (before anti-TB therapy) and at 6 months to quantify inflammatory and coagulation parameters, protein C, and free protein S. Sputum AFB smear was assessed at baseline, 2 months, and 6 months; chest CT was performed at baseline and 6 months. Propensity score matching (age, sex, BMI, smoking) and multivariable regression were used to account for confounding. Logistic regression and ROC analyses evaluated the prediction of BK persistence. **Results**: Compared with controls, TB patients had substantially lower baseline protein C and free protein S levels, and higher D-dimer levels (all *p* < 0.001). In matched multivariable models, TB status remained independently associated with lower baseline natural anticoagulant levels. Lower baseline protein C and free protein S clustered with higher inflammatory markers and higher bacillary burden, and independently predicted BK persistence at 2 and 6 months (OR per 1%-point increase ~0.93–0.95 for protein C and ~0.92–0.94 for free protein S; all *p* < 0.001). Discrimination for BK persistence was high (AUCs ~0.88–0.89). Lower baseline levels of natural anticoagulants were also associated with greater residual CT abnormalities at 6 months. PE cases had significantly lower protein C and free protein S than PE-free patients. **Conclusions:** Active pulmonary TB is associated with marked depletion of protein C and free protein S. Baseline reductions identify patients with higher inflammatory/coagulation activation, higher bacillary burden, delayed microbiological clearance, more residual CT disease, and PE, supporting their potential role as adjunct risk-stratification biomarkers.

## 1. Introduction

Tuberculosis (TB) remains a major global public health concern, with a renewed increase in reported cases after the COVID-19–related disruption of diagnostic and treatment services [1,2,3]. Within the EU/EEA, Romania continues to be disproportionately affected, particularly among socioeconomically vulnerable populations in whom undernutrition may worsen clinical outcomes [4,5]. The burden is amplified in socioeconomically vulnerable populations, where malnutrition/undernutrition can weaken host immunity and worsen disease outcomes [6,7,8]. Global initiatives, including the WHO End TB Strategy, aim to achieve major reductions in TB incidence and mortality by 2035 [9,10].

Beyond pulmonary infection, active TB can trigger systemic inflammation and coagulation disturbances. Proinflammatory mediators, such as IL-1, IL-6, and TNF-α, promote a prothrombotic milieu characterized by increased procoagulant activity—such as changes in PT/aPTT, fibrinogen, and D-dimers—reduced endogenous anticoagulants— such as antithrombin III, protein C, and protein S—and impaired fibrinolysis [11,12]. This imbalance is associated with an increased risk of venous thromboembolism, including deep vein thrombosis and pulmonary embolism, and may adversely affect prognosis and treatment response [13,14,15].

Protein C and free protein S are central anticoagulant regulators with additional immunomodulatory functions. Reduced protein C activity has been reported in TB, potentially reflecting inflammation-related endothelial dysfunction, consumption, and vascular leakage. Reports on free protein S are less consistent, with proposed mechanisms including liver dysfunction and increased binding to complement factors [11,12].

Within this context, free proteins S and C are of particular interest, as they are key anticoagulant regulators with dual functions in modulating coagulation and host immune responses. Given that TB is a chronic infectious disease marked by complex immune and coagulative disturbances, investigating the roles of these proteins may provide new insights into the mechanisms underlying TB-associated thrombosis and systemic immune dysregulation [16,17].

Although reduced protein C and free protein S can occur in inflammatory and malignant states, their clinical significance as risk-stratification biomarkers within confirmed pulmonary TB is not well defined; moreover, despite frequent reporting of routine inflammatory and coagulation markers such as CRP, fibrinogen, D-dimers, prospective data clarifying the prognostic value of natural anticoagulant depletion particularly protein C and free protein S in relation to microbiological response, radiological recovery, and pulmonary embolism remain limited.

However, prospective evidence is limited on whether depletion of natural anticoagulants, specifically protein C and free protein S, adds prognostic information for microbiological conversion, CT radiologic recovery, and pulmonary embolism risk in confirmed pulmonary TB.

Therefore, we prospectively evaluated baseline protein C and free protein S in bacteriologically confirmed pulmonary TB and tested their associations with sputum AFB persistence, CT evolution at 6 months, and clinically suspected pulmonary embolism.

## 2. Materials and Methods

### 2.1. Study Design and Population

This study was conducted prospectively to examine the involvement of free protein S and protein C in tuberculosis, their association with thromboembolic complications, and the relationship between alterations in these anticoagulant pathways and disease severity among patients with confirmed pulmonary TB.

A total of 93 participants were enrolled, comprising 63 patients with bacteriologically confirmed pulmonary TB who were admitted to the Department of Pneumology at Victor Babes University Hospital in Craiova, Romania, between October 2024 and December 2025, and 30 control individuals without pulmonary TB or a history of TB.

This research was conducted in accordance with the principles outlined in the Helsinki Declaration of 1975 and approved by the Ethics Review Board of the University Medicine and Pharmacy of Craiova (No. 408/20.11.2024) and Victor Babes University Hospital (No. 15525/30.10.2024).

Participants in the study were enrolled following voluntary informed consent, without any form of political, social, or religious discrimination, and in full accordance with the data protection legislation.

### 2.2. Inclusion and Exclusion Criteria

The stated aim of the inclusion criteria was to recruit patients aged 18 years or older who provided written informed consent and were bacteriologically confirmed with pulmonary TB. This study only included new cases of pulmonary tuberculosis. TB was defined as having at least one sputum test with a positive acid-fast bacillus test and a positive Xpert MTB RIF test. A new case is that of a patient who had never taken a combination of TB drugs for more than one month.

**Figure 1:**
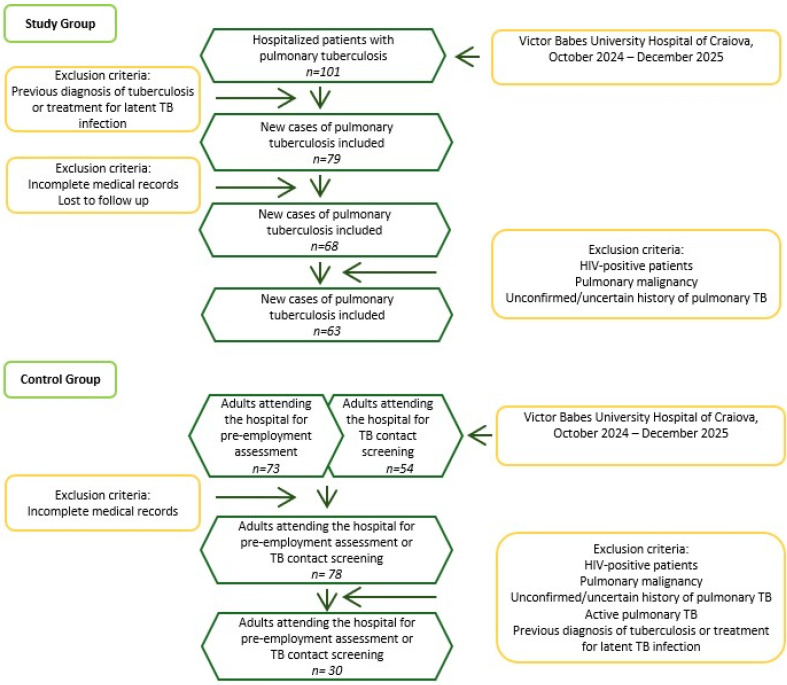
Description of the criteria used to determine the final cohort of study participants.

Initially, a cohort of 101 hospitalized patients with pulmonary tuberculosis was considered, with subsequent focus on those with bacteriological confirmation (*n* = 63). This study included only new cases of pulmonary tuberculosis, as computed tomography (CT) imaging was used to assess the severity of the condition, and previous relapse may have been resolved by pulmonary fibrotic lesions (Figure 1).

All TB cases included in this study were drug-sensitive and had completed the standard six-month treatment regimen recommended by the Romanian National Tuberculosis Control Program. The regimen comprised a two-month intensive phase of daily isoniazid, rifampicin, pyrazinamide, and ethambutol, followed by a four-month continuation phase with isoniazid and rifampicin.

The exclusion criteria included individuals with significant comorbidities, such as chronic respiratory diseases like interstitial lung disease, pulmonary fibrosis, bronchiectasis, asthma, chronic obstructive pulmonary disease, and malignancy; those with a past diagnosis of pulmonary tuberculosis; and those who received treatment for latent TB infection, which could significantly impact the research results. We also excluded HIV-positive patients and those lost to follow-up. Individuals who lacked comprehensive medical records were excluded.

To establish a comparative baseline and isolate the effects of protein C and free protein S on clinical outcomes, we included a control cohort of adults attending the hospital for TB contact screening or pre-employment assessment during the same period who neither had a diagnosis of TB nor a prior history of TB. Control participants were adults (age ≥ 18 years) evaluated at the same institution during the same study period for either TB contact screening or routine pre-employment assessment. All controls provided written informed consent and had no current or prior diagnosis of TB, no history of TB treatment, and no evidence suggestive of active TB at enrollment. Individuals with incomplete medical records were excluded. To minimize confounding by alternative causes of inflammation-driven hypercoagulability, control exclusion criteria mirrored those of the TB group for conditions expected to affect coagulation parameters, including pulmonary malignancy, chronic respiratory diseases such as COPD, asthma, bronchiectasis, interstitial lung disease, pulmonary fibrosis, HIV infection, and incomplete records.

### 2.3. Follow-Up

In alignment with the guidelines of the National Tuberculosis Control Program in Romania, follow-up evaluations were conducted at two, four, and six months following the initial diagnosis. These assessments are designed to monitor patient progress and detect any changes in their health status. The evaluations included serial bacteriological and biological examinations, enabling a comparative analysis across these time points. A CT scan was performed at baseline and again after six months of treatment. The CT data were independently reviewed by two radiologists.

### 2.4. Data Collection

Upon admission, a comprehensive medical history and physical examination were performed. Demographic data, TB risk factors (prior TB, close contact exposure), comorbidities (HIV infection, diabetes mellitus, chronic liver/kidney disease), and smoking history were recorded. Vital signs and anthropometrics (height, weight, body mass index) were measured.

### 2.5. Laboratory Diagnosis of Tuberculosis

Acid-fast bacilli (AFB) sputum examination was conducted microscopically at baseline and repeated at 2 months and 6 months of treatment. Two identification methods were used in this study. Sputum examination was conducted using three specimens of early morning sputum obtained from each patient. The identification of pulmonary TB was contingent upon the utilization of the Ziehl–Neelsen staining method, which entailed the detection of (AFB) through microscopic examination under light/bright-field microscopy and nucleic acid amplification testing. Additionally, a nucleic acid amplification test (GeneXpert, Cepheid, Sunnyvale, CA, USA) using the Xpert MTB/RIF kit was performed to detect Mycobacterium tuberculosis complex. BK persistence was defined as AFB smear positivity on sputum microscopy at the scheduled follow-up visit (2 months and/or 6 months).

Sputum smear microscopy results were graded according to the internationally accepted WHO/IUATLD grading system: fewer than 30 colonies were classified as positive 1–9 AFB; 30–100 colonies were classified as positive 1+, more than 100 isolated colonies were classified as positive 2+, and uncountable confluent colonies were classified as positive 3+.

### 2.6. Measurement of the Biological Serum Parameters

Venous blood samples (6 mL per patient) were collected at baseline and at admission, before initiation of anti-tuberculosis therapy (after an overnight fast). They were repeated at 6 months of treatment and analyzed to determine the complete blood count (leukocytes, hemoglobin, and platelets) using a Alinity automated blood analyzer (Abbott Diagnostics, Abbott Park, Lake County, IL, USA), hepatic enzymes, urea, serum creatinine, and inflammatory markers such as fibrinogen and C-reactive protein (CRP) using an automated blood chemistry analyzer Architect C8000 (Abbott Diagnostics, Abbott Park, Lake County, IL, USA). For coagulation and natural anticoagulant measurements, blood was collected in sodium citrate tubes and processed to platelet-poor plasma according to the manufacturer’s instructions.

### 2.7. Measurement of Serum Levels of Protein C and Free Protein S

Serum concentrations of protein C and free protein S were determined using standardized immunoassays on IL Coagulation Systems (Instrumentation Laboratory, Bedford, MA, USA).

Protein C levels were measured using the HemosIL Protein C chromogenic assay (Cat. No. 0020300500). This automated two-stage assay involves incubation of patient plasma with a protein C activator derived from *Agkistrodon contortrix contortrix* venom, followed by quantification of activated protein C using the synthetic chromogenic substrate S-2366 (pyroGlu-Pro-Arg-pNA·HCl). The paranitroaniline released is monitored kinetically at 405 nm and is directly proportional to the protein C activity in the sample.

Free protein S levels were measured using the HemosIL Free Protein S latex immunoassay (Cat. No. 0020002700). This automated assay utilizes latex agglutination technology in which purified C4b-binding protein (C4BP), adsorbed onto latex particles, binds free protein S from patient plasma in the presence of Ca^2+^ ions. Subsequently, a second latex reagent sensitized with monoclonal antibodies against human protein S triggers agglutination. The degree of turbidity, measured photometrically, is directly proportional to the free protein S concentration in the test sample.

Results for both assays are expressed as percentages of normal activity (% activity for protein C; % normality for free protein S).

### 2.8. Computed Tomography Evaluation and Pulmonary Embolism Assessment

Chest CT was performed systematically in all pulmonary TB patients at baseline (at treatment initiation) and repeated at 6 months, at the end of therapy, in accordance with the Romanian National Tuberculosis Program recommendations, to document radiological evolution under treatment. Baseline CT was used to document disease distribution and severity, including consolidation, nodules/tree-in-bud pattern, and cavitation. In contrast, the 6-month CT (end of treatment) was used to assess radiological response and residual structural sequelae. Pulmonary embolism was not systematically screened; CT pulmonary angiography was performed only in patients with clinical suspicion of PE, and PE was defined as an intraluminal filling defect within the pulmonary arterial tree. All confirmed PE cases were treated according to the standard of care under cardiology guidance, with anticoagulation and follow-up individualized to clinical severity.

### 2.9. Statistical Analysis

Discrete variables were expressed as percentages, while continuous variables were summarized as means with standard deviations. Propensity score matching (PSM) was performed to reduce confounding and balance baseline characteristics between TB patients and controls. PSM is an observational balancing approach that matches/weights participants on the probability of group assignment (TB vs. control) given baseline covariates, thereby reducing confounding when comparing groups.

Propensity scores were estimated using probit regression with age, sex, BMI, and smoking status; matching used a normal kernel with bandwidth 0.03 and common support. Post-matching, regression models were used to estimate the association between TB and protein C/free protein S while adjusting for fibrinogen, CRP, PT, INR, and D-dimers. The average treatment effect on the treated (ATT) and Cohen’s d were reported.

To minimize confounding, we used propensity score matching (with free protein S and protein C as outcomes, respectively) and a kernel method on tuberculosis status, controlling for age, sex, BMI, and smoking status, to improve balance between the TB and control groups. We used a normal kernel with a bandwidth of 0.03. Matching was performed on 93 observations, of which 63 were treated (TB patients) and 30 were controls (TB-free). After matching, all observations were included in the final analysis.

Post-matching, univariate and multivariate regression analyses were conducted to evaluate the associations between TB status and free protein S and protein C levels, adjusting for potential confounders, including fibrinogen, CRP, PT, INR, and D-dimers. The average treatment effect on the treated (ATT) was calculated. Effect sizes were estimated using Cohen’s d. All analyses were performed using Stata software—17.SE-Standard Edition (StataCorp LLC, College Station, TX, USA). Statistical significance was set at *p* < 0.05.

## 3. Results

A total of 93 participants were included in the analysis, comprising 63 patients with active pulmonary TB and 30 control individuals without a history of TB. Baseline demographic, clinical, biochemical, and hematological parameters were compared between the two groups to identify disease-related alterations.

### 3.1. Comparison of Biological Parameters Between TB Patients at Baseline and Controls

The two groups were comparable in terms of age, sex distribution, smoking status, and pack-years, with no statistically significant differences observed (Table 1).

Compared with controls, TB patients had lower BMI and albumin and markedly higher inflammatory markers (ESR, CRP, fibrinogen) (Table 1). Coagulation findings were consistent with a hypercoagulable profile, including higher D-dimers, alongside elevated transaminases; renal function markers were largely similar between groups.

### 3.2. Comparison of Biological Parameters Between TB Patients at Baseline and Controls According to Protein Free S and Protein C Levels

#### 3.2.1. Univariate Analysis

The analysis of biological parameters showed clear time-dependent differences when TB patients were stratified by baseline free protein S and protein C levels (Table 2). At baseline, patients with low free protein S displayed a distinctly more inflammatory and prothrombotic biochemical profile than those with normal free protein S, characterized by higher ESR, CRP, and fibrinogen levels, higher platelet counts and D-dimers, and lower hemoglobin levels. These contrasts were statistically significant and accompanied by large effect sizes, consistent with pronounced inflammation, anemia, and reactive thrombocytosis in patients with reduced natural anticoagulant activity.

A similar baseline pattern was observed after stratification by protein C. Patients with low protein C had markedly higher inflammatory markers and D-dimers, lower hemoglobin, and higher platelet counts compared with those with normal protein C, again indicating a more severe inflammatory/hematologic phenotype at presentation in the low anticoagulant subgroup.

By 6 months, these between-group differences disappeared. Inflammatory markers, platelet counts, and D-dimers declined to low levels, and hemoglobin improved in both strata, with only small, non-significant differences between the low vs. normal free protein S groups and the low vs. normal protein C groups. Collectively, these results support that reduced free protein S or protein C at baseline is associated with a markedly disturbed inflammatory and coagulation profile at diagnosis. However, this separation is not sustained at follow-up.

Table 3 shows a clear time point-dependent pattern. At baseline, both free protein S and protein C were strongly and inversely correlated with smear and culture load, indicating that lower natural anticoagulant levels at presentation are associated with a higher mycobacterial burden. In contrast, baseline radiologic extent on CT showed only a modest inverse association with free protein S and no clear association with protein C.

When baseline burden was compared to follow-up anticoagulant levels, higher baseline smear and culture load correlated positively with 6-month free protein S. Meanwhile, correlations with 6-month protein C were weak and non-significant. At 6 months, smear and culture load showed little association with 6-month free protein S, but modest positive correlations emerged with 6-month protein C. Greater radiologic extent at 6 months and higher culture load at 6 months were inversely correlated with baseline free protein S and baseline protein C, suggesting that lower baseline anticoagulant levels are linked to more persistent microbiological burden and more extensive residual radiologic disease at follow-up.

#### 3.2.2. Multivariate Analysis—Propensity Score Matching

Age, sex, BMI, and smoking status, when used together, maintained a reasonably good model fit (Pseudo R^2^ = 0.4526) and balanced some key confounders related to TB status. BMI was strongly associated with tuberculosis, while age and sex, although not statistically significant individually in the probit, helped capture important demographic differences and improve overall balance.

The propensity score-matching results indicate that patients with TB had substantially lower levels of Free S protein and C protein than matched controls. Specifically, the average treatment effect on the treated (ATT) revealed that Free S protein was significantly lower by 38.92 units (*p* < 0.001) in the TB group, and C protein was lower by 26.99 units (*p* < 0.001). These results suggest a robust association between TB disease and reduced levels of natural anticoagulant proteins, underlining the lasting biochemical impact of TB (Table 4).

After propensity score matching, the multivariable models indicate that TB status is independently associated with lower baseline levels of both free protein S and protein C (both *p* < 0.001; Table 5). At baseline, lower free protein S was additionally associated with higher BMI and higher D-dimer concentrations. Meanwhile, age, sex, fibrinogen, CRP, INR, and PT were not independently associated with free protein S. In the baseline protein C model, lower levels were associated with older age and higher D-dimers, whereas higher BMI and longer PT were associated with higher protein C; sex, fibrinogen, CRP, and INR were not significant. At 6 months, the pattern of predictors shifted. BMI remained independently associated with both anticoagulant proteins (lower free protein S and higher protein C), and higher CRP was associated with lower levels of both proteins at follow-up. In contrast, D-dimers and age were no longer significant at 6 months. PT showed a positive association with free protein S at 6 months, but was not associated with protein C.

Both the univariate and multivariate analyses suggest that tuberculosis is associated with a significant decrease in free S protein and C protein levels, suggesting an alteration in immune response or other biological processes related to the disease.

### 3.3. Analysis of Response to TB Treatment in Relation to the Level of Free Protein S and Protein C

Analysis of tuberculosis persistence during treatment revealed a strong association with reduced levels of natural anticoagulant proteins. At two months, persistence of *Koch Bacilli* (BK) in sputum was observed in 60.0% of patients with low free protein S levels, compared with only 4.8% in those with normal levels. Similarly, persistence was present in 55.6% of patients with low protein C compared to just 1.8% with normal protein C. At six months, persistence rates declined but remained significantly higher among patients with low free protein S (16.7% vs. 1.6%) and low protein C (16.7% vs. 0%). Odds ratios demonstrated a markedly increased risk of persistence in patients with low levels of free protein S and protein C at both time points.

Multivariate logistic regression analyses were conducted to assess the associations of baseline levels of C protein and free S protein, gender, smoking status, and underweight status with BK persistence in sputum at 2 months and 6 months. Odds ratios (ORs) and *p*-values are presented in Table 6.

In multivariable logistic regression, each 1%-point increase in baseline protein C was associated with reduced odds of BK-positive sputum at 2 months (OR 0.95, 95% CI [0.93–0.97], *p* < 0.001) and 6 months (OR 0.93, 95% CI [0.89–0.96], *p* < 0.001) (Table 5). Underweight status was associated with increased odds of persistence at 2 months (OR 4.75, CI [1.65–13.69], *p* = 0.004) and showed a similar trend at 6 months (OR 4.51, CI [0.86–23.69], *p* = 0.075). Sex and smoking were not significant predictors.

Similarly, each 1%-point increase in baseline free protein S was associated with reduced odds of BK persistence at 2 months (OR 0.94, *p* < 0.001) and 6 months (OR 0.92, *p* < 0.001) (Table 7). Underweight status was associated with increased odds at both time points (2 months: OR 4.73, *p* = 0.005; 6 months: OR 3.90, *p* = 0.047), while sex and smoking were not significant predictors.

**Figure 2:**
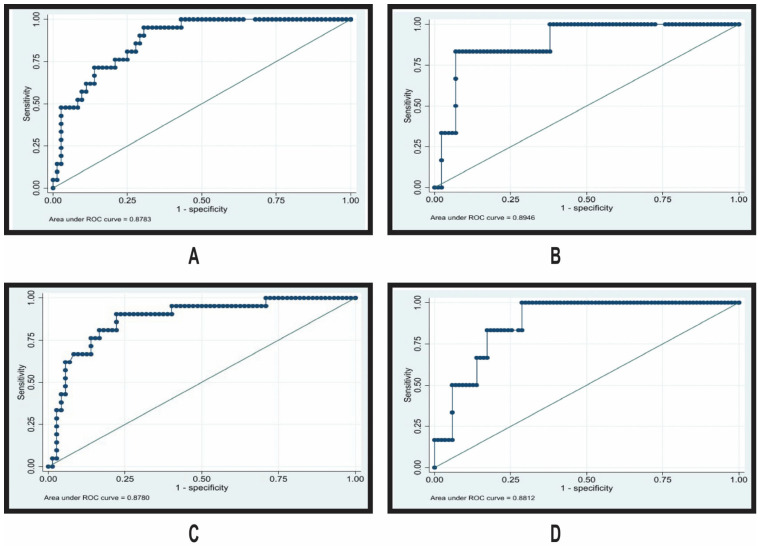
Receiver operating characteristic (ROC) curves for the prediction of tuberculosis persistence using baseline natural anticoagulant protein levels. BK: *Koch Bacilli*; AUC: area under the curve. (**A**) Protein C predicting BK persistence at 2 months of treatment (AUC = 0.8946). (**B**) Protein C predicting BK persistence at 6 months of treatment (AUC = 0.8812). (**C**) Free protein S predicting BK persistence at 2 months of treatment (AUC = 0.8783). (**D**) Free protein S predicting BK persistence at 6 months of treatment (AUC = 0.8780).

Receiver operating characteristic (ROC) curves were generated to evaluate the discriminative ability of baseline free protein S and C levels in predicting tuberculosis persistence during treatment (Figure 2).

ROC analysis showed good discrimination of baseline protein C and free protein S for predicting BK persistence at 2 and 6 months (protein C AUC 0.8946 and 0.8812; free protein S AUC 0.8783 and 0.8780). 

All ROC curves demonstrated steep initial rises and maintained high sensitivity across most specificity thresholds, suggesting these biomarkers could serve as effective screening tools. The curves are consistently positioned well above the diagonal reference line, confirming that their predictive value substantially exceeds chance.

All curves demonstrate excellent discriminative ability with AUC values >0.87. The diagonal reference line represents an AUC of 0.50 (no discrimination). Higher baseline levels of both protein C and free protein S were associated with lower odds of BK persistence at both time points.

### 3.4. Imaging CT

Radiological findings stratified by baseline protein C were broadly similar at baseline, with tree-in-bud opacities, consolidations, and cavities distributed across lobes and no significant differences at most sites. The main baseline difference was more frequent cavitation in the superior left lobe among patients with low protein C (63.9% vs. 22.2%, *p* = 0.001), with non-significant trends toward more infiltrates (19.4% vs. 3.7%, *p* = 0.063) and consolidations (33.3% vs. 14.8%, *p* = 0.095) in the inferior left lobe.

At 6 months, residual abnormalities were more pronounced in the low protein C group, including higher rates of bronchiectasis in the middle right lobe (13.9% vs. 0%, *p* = 0.043), fibrosis in the inferior left lobe (16.7% vs. 0%, *p* = 0.025), and nodular consolidations in the superior (50% vs. 3.7%, *p* < 0.001) and inferior left lobes (13.9% vs. 0%, *p* = 0.043), with additional non-significant trends for fibrosis in the superior left lobe (44.4% vs. 22.2%, *p* = 0.067) and middle right lobe (22.2% vs. 0%, *p* = 0.089).

Notably, the complete absence of radiological lesions at six months was documented in one-third of patients with normal protein C but in none of those with low protein C (*p* < 0.001), underscoring a differential pattern of radiological recovery linked to baseline protein C status.

Radiological assessment at baseline revealed distinct patterns between patients with normal and low free protein S levels. While tree-in-bud opacities were frequent across both groups, they did not differ significantly in most lobes, except for the inferior right lobe, where such findings were observed exclusively among patients with low free protein S (10% vs. 0%, *p* = 0.063, trend). Infiltrative changes in the inferior left lobe were significantly more common in patients with low free protein S (23.3% vs. 3.0%, *p* = 0.016). Similarly, consolidations were more frequent in the superior left lobe (73.3% vs. 48.5%, *p* = 0.044) and the inferior left lobe (40.0% vs. 12.1%, *p* = 0.011) among those with reduced free protein S. Cavitary lesions showed a striking difference in the superior left lobe, present in two-thirds of low free protein S patients compared with just over a quarter of those with normal levels (66.7% vs. 27.3%, *p* = 0.001).

At six months of treatment, patients with low free protein S continued to exhibit a higher burden of radiological sequelae. Bronchiectasis was significantly more frequent in the middle right lobe (16.7% vs. 0%, *p* = 0.014). Fibrotic changes were markedly more common in the middle right (26.7% vs. 0%, *p* = 0.001), superior left (53.3% vs. 18.2%, *p* = 0.003), and inferior left lobes (20.0% vs. 0%, *p* = 0.007). Nodular consolidations were also significantly more frequent in the middle right (33.3% vs. 9.1%, *p* = 0.017), superior left (50.0% vs. 12.1%, *p* = 0.001), and inferior left lobes (16.7% vs. 0%, *p* = 0.014) in the low free protein S group.

Importantly, the complete absence of radiological lesions at six months was documented in more than a quarter of patients with normal free protein S but in none with low levels (27.3% vs. 0%, *p* = 0.002), underscoring impaired radiological recovery in the latter group.

### 3.5. Free Protein S and Protein C Levels in Relation to Thromboembolic Events

Patients with PE had significantly lower levels of free protein S and protein C than PE-free patients. The differences were statistically significant and associated with large effect sizes (free protein S: *p* = 0.010, Cohen’s d = 0.91; protein C: *p* = 0.001, Cohen’s d = 1.14). In contrast, comparisons according to stroke status did not reach statistical significance. These analyses were severely underpowered due to the very small number of stroke cases (*n* = 2) and should be interpreted as exploratory (Table 8).

## 4. Discussion

This study demonstrates that, despite comparable demographic and lifestyle characteristics, patients with active TB exhibited profound systemic alterations affecting nutritional, hematological, inflammatory, and coagulation parameters.

The significantly lower BMI and hypoalbuminemia observed among TB patients are consistent with the well-documented catabolic state and protein-energy malnutrition associated with chronic infection. Malnutrition has been shown to increase susceptibility to TB and impair immune responses, creating a vicious cycle that worsens prognosis [18,19].

The pronounced elevation of ESR, fibrinogen, and CRP highlights the robust inflammatory response induced by *Mycobacterium tuberculosis*. Elevated inflammatory markers are hallmarks of active TB and have been correlated with disease severity in multiple studies [20,21]. The reduction in hemoglobin is consistent with anemia of chronic disease, which is frequently reported in TB and is linked to cytokine-mediated suppression of erythropoiesis [22].

The hematological profile was further characterized by thrombocytosis, a finding common in TB and reflecting systemic immune activation [23].

Coagulation abnormalities emerged as a striking feature. Shortened clotting times, elevated prothrombin index, and markedly increased D-dimers indicate a hypercoagulable state. Similar prothrombotic profiles have been reported in TB and are thought to reflect systemic inflammation, including elevated ESR/CRP/fibrinogen, endothelial dysfunction, and increased fibrin turnover [24,25]. The significant reduction in protein C and free protein S levels further supports the presence of impaired anticoagulant pathways, predisposing patients to thromboembolic events. Previous reports have emphasized the heightened risk of venous thromboembolism in TB patients, underscoring the clinical relevance of these findings [26]. One study directly measured protein C levels in tuberculosis patients. It found that active TB is associated with significantly lower protein C activity compared to latent TB and healthy controls. Protein C levels increased after treatment, returning to normal in latent TB and post-treatment groups [13]. The significant alterations in natural anticoagulant pathways, as evidenced by markedly reduced serum levels of free protein S and protein C in affected patients, were consistently observed across univariate, multivariate, and propensity score–matched analyses, underscoring their robustness and potential clinical relevance.

Patients with low free protein S and C levels exhibited more pronounced systemic inflammation, as reflected by higher ESR, CRP, and fibrinogen concentrations. These findings are in line with the recognized role of chronic infection and immune activation in promoting a prothrombotic state. Elevated inflammatory markers have been shown to reduce anticoagulant protein activity by impairing hepatic synthesis and increasing consumption during ongoing coagulation activation [27]. The marked anemia observed in patients with low free protein S and protein C may further reflect systemic inflammation and nutritional deficiencies, both of which are commonly associated with TB [22].

The significantly elevated platelet counts in patients with reduced free protein S and C reinforce the concept of a hypercoagulable state. Thrombocytosis in TB has been previously described and attributed to elevated interleukin-6 and other cytokines that drive megakaryopoiesis [28]. Our findings align with prior observations linking elevated platelet counts to heightened thrombotic risk in chronic infections. The concurrent increase in D-dimer levels among patients with low anticoagulant proteins further supports ongoing fibrin turnover and subclinical coagulation activation [29].

Robson et al. reported that TB patients exhibit reduced levels of natural anticoagulants and increased thrombin generation, proposing that the disease induces a systemic prothrombotic response [25]. Similarly, Ambrosetti et al. highlighted the increased incidence of venous thromboembolism in TB populations, emphasizing the contribution of infection-related coagulation abnormalities [24]. More recent studies have also confirmed that reductions in protein C and free protein S are common in chronic inflammatory diseases, where they may serve as biomarkers of thrombotic risk [30].

Nutritional recovery was further reflected by increases in serum total protein and albumin. Hypoalbuminemia is frequently observed in TB and correlates with disease severity [21]. By 6 months of treatment, the inflammatory and coagulation-activation phenotype present at diagnosis had largely resolved. Inflammatory markers and reactive hematologic changes improved substantially, with recovery of hemoglobin and normalization of platelet counts, alongside a marked decline in D-dimers, consistent with reduced fibrin turnover and attenuation of systemic coagulation activation. Overall, these findings support that TB-associated hypercoagulability is closely linked to active inflammatory disease and is largely reversible with effective therapy. Meanwhile, baseline depletion of natural anticoagulants remains clinically relevant given its association with bacillary burden and delayed microbiological and radiologic recovery [15,25]. The observed recovery of protein C and free protein S further supports this, emphasizing their role as biomarkers of thrombotic risk and treatment response [12].

Across analyses, reduced natural anticoagulant levels at diagnosis clustered with a more severe systemic disease phenotype. Patients with low free protein S or low protein C showed a pronounced inflammatory–hematologic profile at baseline, with elevated inflammatory markers and coagulation activation, as well as anemia and thrombocytosis. These differences were largely attenuated by 6 months with treatment. In parallel, baseline free protein S and protein C were inversely correlated with baseline bacillary burden (smear and culture load), supporting that depletion of natural anticoagulants tracks mycobacterial burden more closely than CT extent at presentation [31,32].

Baseline anticoagulant status is also related to follow-up disease status. Lower baseline free protein S and protein C correlated with higher culture load and greater radiologic extent at 6 months, suggesting that early depletion of these proteins identifies patients with slower microbiological and radiologic resolution during therapy. This pattern is compatible with the established link between inflammation-driven hypercoagulability and TB severity, in which reduced protein C/S may reflect inflammatory consumption and/or impaired hepatic synthetic capacity in more advanced disease rather than a beneficial inflammatory response. These findings partially align with the existing literature. Prior studies have shown that high levels of CRP and other acute-phase/inflammatory biomarkers correlate with disease severity and bacterial burden at the time of pulmonary tuberculosis diagnosis. For instance, Kivrane et al. (2024) observed that lower BMI, positive sputum smear, and lung cavitation were associated with higher baseline CRP levels and with slower reductions in CRP after treatment initiation [33]. Similarly, studies using CRP as a triage or screening tool show that elevated CRP is common in more severe or advanced TB disease [34].

The multivariate analysis provides additional insights into the determinants of free protein S and C levels. Beyond TB status, both age and D-dimer concentrations were negatively associated with these anticoagulant proteins, while BMI showed variable effects. These results are biologically plausible: advancing age and increased fibrin turnover have been linked to reductions in natural anticoagulant activity [27,29]. Meanwhile, low BMI in TB likely reflects malnutrition, which impairs hepatic protein synthesis [35].

In multivariable models after propensity score matching, TB status remained independently associated with lower baseline free protein S and protein C. Meanwhile, D-dimers and BMI were independently associated with anticoagulant levels (with directions differing by protein and time point). These reinforce that these markers integrate systemic inflammation, coagulation activation, and host factors. Taken together, baseline protein C and free protein S may serve as pragmatic adjunct biomarkers for risk stratification, identifying patients more likely to have a higher burden at diagnosis and more residual microbiological or radiological disease at follow-up. Some prior work, however, suggests that high baseline CRP predicts poorer outcomes or delayed sputum conversion; for example, elevated CRP is often associated with smear positivity, cavitary disease, and greater bacillary burden, all of which are themselves factors for delayed conversion [33,36].

Our results are consistent with earlier studies showing that TB is associated with a hypercoagulable state and reduced natural anticoagulants [27], which may contribute to impaired pathogen clearance and slower clinical recovery [15,17]. The absence of persistence at six months in patients with normal protein C highlights the potential utility of these biomarkers in identifying high-risk subgroups who may benefit from closer monitoring or adjunctive interventions.

Radiologic findings in our cohort suggest that baseline natural anticoagulant status is linked to the subsequent radiologic trajectory of pulmonary TB under treatment. At diagnosis, CT disease extent (defined as the number of lobes with TB-specific lesions) showed only a modest inverse association with free protein S and no clear association with protein C, indicating that baseline imaging extent is not strongly captured by protein C levels alone. However, greater CT extent at 6 months was consistently associated with lower baseline levels of both protein C and free protein S, suggesting that early depletion of these anticoagulant proteins identifies patients with more persistent radiologic involvement during therapy. Taken together with the parallel associations between baseline anticoagulant depletion and higher bacillary burden, these findings support a model in which reduced protein C and free protein S reflect a systemic inflammatory coagulation phenotype accompanying more severe disease and slower radiologic resolution. This aligns with prior literature showing that persistent or extensive radiologic disease is prognostically important in TB [17,25], and with emerging evidence that inflammation-driven hypercoagulability may contribute to tissue injury and impaired repair mechanisms [37].

In this study, pulmonary embolism was associated with significantly lower circulating levels of free protein S and protein C, with large standardized effect sizes. These findings are consistent with the established role of the protein C-free protein S anticoagulant pathway in the regulation of thrombin generation and venous thromboembolism pathophysiology [38,39]. Previous studies have repeatedly demonstrated that deficiencies, whether inherited or acquired, of these natural anticoagulants are strongly linked to venous thromboembolic events, particularly pulmonary embolism. The magnitude of the observed differences in our cohort is comparable to those reported in studies evaluating protein C and free protein S abnormalities in patients with acute or recent venous thromboembolism, supporting the biological plausibility of our results [17].

However, interpreting reduced protein C and free protein S levels in the setting of acute pulmonary embolism requires caution. It is well recognized that these proteins may be transiently reduced during acute thrombotic events, inflammatory states, or under anticoagulant therapy, particularly vitamin K antagonists [12]. As measurements in the present study were obtained in a clinical context rather than during a stable baseline state, the observed associations cannot distinguish between pre-existing thrombophilia and secondary, event-related reductions. Consequently, our findings should be interpreted as demonstrating an association between pulmonary embolism and lower protein C and free protein S levels, rather than as evidence of causality or inherited deficiency.

In contrast to pulmonary embolism, no statistically significant associations were observed between protein C or free protein S levels and stroke. This result is not unexpected given the extremely small number of stroke cases in the cohort (*n* = 2), which severely limits statistical power and precision. Moreover, the relationship between deficiencies of natural anticoagulants and arterial thrombotic events such as ischemic stroke is less consistent in the literature than for venous thromboembolism, with reported associations often restricted to younger patients or specific clinical scenarios [40,41]. In this context, the stroke-related analyses in the present study should be regarded as exploratory and descriptive only.

Our findings show that acute pulmonary embolism is accompanied by pronounced reductions in circulating free protein S and protein C, underscoring the physiological importance of the protein C-free protein S anticoagulant pathway in venous thromboembolism. These observations emphasize that clinical interpretation of protein C and free protein S levels must account for sampling timing, acute-phase conditions, and ongoing treatment, and they underline the need for larger, prospective studies with standardized sampling protocols.

## 5. Limitation

This prospective study has several limitations. First, it was conducted at a single center, which may limit the generalizability of the findings beyond similar settings and care pathways. Second, although propensity score methods were used to mitigate confounding, residual and unmeasured confounding, such as micronutrient/vitamin K status, detailed dietary intake, subclinical liver dysfunction, and inflammatory comorbidities, cannot be excluded. Because CT pulmonary angiography was performed when PE was clinically suspected (no routine screening), the incidence of PE in this cohort may be underestimated. Third, the CTs were obtained at baseline and at month 6; they were not obtained at intermediate time points. Lastly, although two radiologists read the scans independently, we did not use a standardized semi-quantitative scoring system or formal inter-rater reliability metrics.

## 6. Future Direction

Future studies should validate these findings in larger, multicenter cohorts, incorporate standardized CT severity scoring, and assess whether protein C/free protein S provides incremental predictive value beyond smear grade and radiologic extent. Mechanistic studies integrating endothelial and cytokine markers may clarify causal pathways. Interventional studies could explore whether targeted thromboprophylaxis strategies are beneficial in high-risk TB subgroups.

## 7. Conclusions

In this prospective cohort, active drug-susceptible pulmonary TB was associated with significant reductions in protein C and free protein S, along with a hyperinflammatory and hypercoagulable profile. Lower baseline natural anticoagulant levels were associated with higher bacillary burden and independently predicted BK persistence at 2 and 6 months, with good discriminative performance on ROC analysis. Baseline depletion was also linked to poorer radiologic recovery at 6 months and was associated with pulmonary embolism in clinically suspected cases. These findings support evaluating protein C and free protein S as pragmatic adjunct biomarkers for risk stratification in confirmed pulmonary TB. Meanwhile, larger multicenter studies are needed to confirm generalizability and clarify the temporal and causal relationships.

## Figures and Tables

**Table 1:** Differences in baseline biological parameters between TB patients and controls.

Variable	Control	TB Patients	*p* Value
Age	45.50 ± 12.68	45.63 ± 12.92	0.962
Urban residence	76.67%	79.37%	0.767
Male gender	80.00%	80.95%	0.913
Smoker	60.00%	58.73%	0.907
Packs/year	10.70 ± 10.79	10.78 ± 10.13	0.973
BMI	23.20 ± 02.59	18.77 ± 02.01	<0.001
ESR (0–20 mm/h)	05.23 ± 02.06	68.68 ± 41.43	<0.001
FIB (200–400 mg/dL)	299 ± 65	525 ± 150	<0.001
CRP (0–5 mg/L)	02.93 ± 01.43	97.63 ± 52.59	<0.001
HB (12–18 g/dL)	14.81 ± 01.72	11.69 ± 01.70	<0.001
WBC (4–10×10^3^/µL)	7198 ± 1559	9743 ± 2657	<0.001
NEU (30–70%)	3710 ± 1476	4337 ± 1814	0.103
PLT (150–450×10^3^/µL)	277,517 ± 85,274	545,830 ± 192,335	<0.001
EOS (0–5%)	47.34 ± 37.51	221.25 ± 128.72	<0.001
Uric acid (2.4–5.7 mg/dL)	04.08 ± 01.13	04.91 ± 01.46	0.007
AST (0–35 U/L)	22.78 ± 08.56	48.84 ± 23.96	<0.001
ALT (0–35 U/L)	26.78 ± 14.73	48.19 ± 19.85	<0.001
Urea (16.6–48.5 mg/dL)	30.91 ± 07.70	26.44 ± 09.44	0.026
Creatinine (0.5–0.9 mg/dL)	00.69 ± 00.11	00.90 ± 01.05	0.280
Total Protein (64–83 g/L)	73.75 ± 05.59	61.29 ± 05.85	<0.001
Albumin (35–52 g/L)	43.45 ± 04.73	30.58 ± 06.22	<0.001
C Protein (70–140%)	97.32 ± 20.44	66.44 ± 12.10	<0.001
Free S Protein (54.7–123.7%)	87.40 ± 21.56	55.85 ± 14.00	<0.001
INR (0.4–1.3)	00.91 ± 00.28	00.73 ± 00.21	0.002
APTT (22–38 s)	30.64 ± 04.86	24.23 ± 06.71	<0.001
PT (10–15 s)	12.48 ± 01.40	10.39 ± 02.43	<0.001
Prothrombin index (80–120%)	102 ± 12	115 ± 17	0.001
D-Dimers (0–0.5 µg/mL)	0.25 ±0.15	3.07 ± 2.38	<0.001

BMI: Body mass index; ESR: Erythrocyte sedimentation rate; FIB: fibrinogen; CRP: C-reactive protein; HB: Hemoglobin; WBC: White blood cell; NEU: Neutrophil; PLT: Platelet; EOS: Eosinophil; AST: Aspartate aminotransferase; ALT: Alanine aminotransferase; INR: International normalized ratio; APTT: Activated partial thromboplastin time; PT: Prothrombin time.

**Table 2:** Biological parameters at baseline and 6 months in patients with TB according to free protein S and C levels.

		Normal Free S Protein	Low Free S Protein	Cohen’s d	Normal C Protein	Low C Protein	Cohen’s d
ESR (mm/h)	Baseline	45.61 ± 31.18	94.07 ± 36.39	−1.44 *	38.18 ± 20.92	91.56 ± 38.20	−1.67 *
	6 months	8.76 ± 2.75	9.2 ± 2.63	−0.16	8.55 ± 2.81	9.27 ± 2.57	−0.27
CRP (mg/L)	Baseline	65.48 ± 42.93	133 ± 37.54	−1.67 *	57.63 ± 39.11	127.64 ± 40.04	−1.77 *
	6 months	2.02 ± 1.39	2.59 ± 1.54	−0.38	2.08 ± 1.42	2.44 ± 1.52	−0.24
FIB (mg/dL)	Baseline	435.51 ± 140.51	623.93 ± 84.87	−1.6 *	391.48 ± 106.43	625.56 ± 85.98	−2.46 *
	6 months	286.79 ± 59.06	295.00 ± 56.95	−0.14	280.91 ± 58.85	298.04 ± 56.61	−0.3
HB (g/dL)	Baseline	12.67 ± 1.42	10.62 ± 1.30	+1.49 *	13.10 ± 0.95	10.64 ± 1.34	+2.06 *
	6 months	13.68 ± 1.16	13.51 ± 1.10	−0.15	13.73 ± 1.22	13.51 ± 1.06	0.20
PLT (×10^3^/µL)	Baseline	426,303 ± 159,927	677,310 ± 129,326	−1.72 *	367,814 ± 88,169	679,341 ± 130,287	−2.73 *
	6 months	301,650 ± 89,921	303,125 ± 79,192	−0.25	311,277 ± 82,663	303,159 ± 86,674	0.02
D-Dimers (µg/mL)	Baseline	1.58 ± 1.43	4.71 ± 2.13	−1.74 *	1.81 ± 1.19	4.49 ± 2.04	−1.91 *
	6 months	0.27 ± 0.15	0.32 ± 0.22	−0.25	0.29 ± 0.13	0.29 ± 0.22	0.02

ESR: Erythrocyte sedimentation rate; CRP: C-reactive protein; FIB: fibrinogen; HB: Hemoglobin; PLT: platelets. * *p* < 0.05.

**Table 3:** Spearman correlation between baseline and 6 months natural anticoagulant levels (free protein S and protein C) and baseline TB burden and CT radiologic extent.

Parameter		Spearman’s Rho (Free Protein S)		Spearman’s Rho (Protein C)	
		Baseline	6 months	Baseline	6 months
Smear load	Baseline	−0.634 *	0.238 *	−0.695 *	0.086
	6 months	−0.172	0.176	0.154	0.254 *
Culture load	Baseline	−0.677 *	0.316 *	−0.701 *	0.048
	6 months	−0.245 *	−0.084	−0.322 *	0.245 *
Radiologic extent	Baseline	−0.310 *	0.141	−0.201	0.026
	6 months	−0.492 *	0.110	−0.582 *	0.117

Radiologic extent was assessed as the number of lobes in which TB-specific lesions were detected on CT. * *p* < 0.05.

**Table 4:** Propensity score matching results.

Variable	Control (*n* = 30)	TB (*n* = 63)	Mean Difference (ATT)	*p* (ATT)
Free S protein (%)	87.40 ± 21.56	55.85 ± 14.00	−38.92	<0.001
C protein (%)	97.32 ± 20.44	66.44 ± 12.10	−26.99	<0.001

TB: tuberculosis history; ATT: average treatment effect on the treated.

**Table 5:** Multivariate analysis of the impact of risk factors on free protein S and protein C levels after propensity score matching.

	Free Protein S				Protein C			
	Coefficient		*p* > |t|		Coefficient		*p* > |t|	
Time Point	Baseline	6 Months	Baseline	6 Months	Baseline	6 Months	Baseline	6 Months
Variable								
Tuberculosis	−26.96	-	<0.001	-	−17.82	-	<0.001	-
Age (years)	−0.15	−0.13	0.153	0.297	−0.47	−0.08	0.000	0.450
Male gender	0.42	2.14	0.900	0.613	0.81	2.07	0.813	0.545
BMI	−2.13	−2.50	0.040	0.004	2.47	3.44	0.022	<0.001
FIB (mg/dL)	−0.01	−0.03	0.611	0.214	0.02	−0.01	0.268	0.818
CRP (mg/dL)	−0.02	−2.93	0.646	0.01	0.02	−1.80	0.653	0.046
D-dimers (µg/mL)	−3.99	−2.68	0.002	0.754	−3.03	−2.10	0.023	0.762
INR	1.35	3.16	0.810	0.679	−6.60	−6.56	0.256	0.291
PT (s)	−0.42	2.40	0.526	0.03	1.72	0.29	0.013	0.753
Constant	151.97	3.88	0.000	0.879	42.11	10.97	0.104	0.596

BMI: Body mass index; FIB: fibrinogen; CRP: C-reactive protein; INR: International normalized ratio; PT: prothrombin time.

**Table 6:** Multivariate analysis of BK persistence in sputum at 2 months according to the level of C protein.

Variable	BK Persistence at 2 Months		BK Persistence at 6 Months	
	OR	*p* > |z|	OR	*p* > |z|
C protein at baseline (%)	0.95	0.000	0.93	0.000
Male gender	2.71	0.304	2.00	0.564
Smoker	2.11	0.289	2.46	0.397
Underweight	4.75	0.004	4.51	0.075

OR: Odds Ratio.

**Table 7:** Multivariate analysis of BK persistence in sputum at 2 months according to the level of free protein S.

Variable	BK Persistence at 2 Months		BK Persistence at 6 Months	
	OR	*p* > |z|	OR	*p* > |z|
Free S protein at baseline (%)	0.94	0.000	0.92	0.000
Male gender	2.55	0.329	1.28	0.784
Smoker	2.41	0.223	2.31	0.317
Underweight	4.73	0.005	3.90	0.047

OR: Odds Ratio.

**Table 8:** Protein C and free protein S levels at baseline according to PE and stroke status.

Group	Protein C (%)	Free Protein S (%)
PE analysis		
PE-free	68.47 ± 10.79	57.78 ± 13.36
PE	55.63 ± 13.46	45.59 ± 13.44
Mean difference (95% CI)	12.8 (5.1 to 20.6)	12.2 (3.0 to 21.4)
*p* value	0.0015	0.010
Cohen’s d (95% CI)	1.14 (0.43–1.85)	0.91 (0.21–1.60)
Stroke analysis		
Stroke-free	66.91 ± 11.98	56.36 ±13.95
Stroke	52.00 ± 5.66	40.50 ± 2.12
Mean difference (95% CI)	14.9 (−2.2 to 32.0)	15.9 (−4.0 to 35.7)
*p* value	0.086	0.116
Cohen’s d (95% CI)	—	—

Abbreviations: PE, pulmonary thromboembolism; CI, confidence interval. Cohen’s d is reported only where group sizes were sufficient for meaningful estimation.

## Data Availability

The data presented in this study are available upon request from the corresponding author. The data are not publicly available due to the patient’s personal data protection policy of the University and Hospital.
